# Establishment of new predictive markers for distant recurrence of colorectal cancer using lectin microarray analysis

**DOI:** 10.1002/cam4.342

**Published:** 2014-10-30

**Authors:** Kentaro Nakajima, Masafumi Inomata, Hidekatsu Iha, Takahiro Hiratsuka, Tsuyoshi Etoh, Norio Shiraishi, Kenji Kashima, Seigo Kitano

**Affiliations:** 1 Department of Gastrointestinal and Pediatric Surgery, Oita University Faculty of MedicineHasama-machi, Oita, Japan; 2 Department of Microbiology, Oita University Faculty of MedicineHasama-machi, Oita, Japan; 3 Department of Diagnostic Pathology, Oita University Faculty of MedicineHasama-machi, Oita, Japan

**Keywords:** Colorectal cancer, glycoprotein, lectin, prediction, recurrence

## Abstract

We evaluated the clinical benefits of novel predictive markers for distant recurrence with colorectal cancer using lectin microarray analysis of cell surface glycan modifications. Glycoproteins were extracted from formalin-fixed, paraffin-embedded tumor specimens and normal epithelium from 53 consecutive curatively resected stage I–III colorectal cancer cases and then subjected to lectin microarray to obtain lectin–glycan interaction (LGI) values. In addition, clinicopathological factors associated with distant recurrence were identified. LGI values that were associated with distant recurrence were validated with an additional 55 curatively resected stage II colorectal cancer cases. LGI values for *Agaricus bisporus* (ABA) lectin, prominent in cancer tissues, were statistically associated with distant recurrence. ABA lectin staining exhibited strikingly intense signals in the cytoplasm and apical surfaces of cancer cells, while weak staining was observed in the supranuclear regions of normal epithelium. This ABA tumor/normal LGI ratio may be a new predictive biomarker for distant recurrence of curatively resected colorectal cancer.

## Introduction

Colorectal cancer is a major cause of morbidity and mortality, with a worldwide annual incidence that ranks third among men (746,000 cases; 10.0% of total cancers per year) and second among women (614,000 cases; 9.2%) 1. Surgery remains the main curative therapy for colorectal cancer. Adjuvant chemotherapy after complete tumor resection is effective for reducing the recurrence risk 2–4. Although adjuvant chemotherapy is standard care for patients with stage III colon cancer, its role for stage II colon cancer remains controversial 3,5. Thus, determining the optimal indications for adjuvant chemotherapy, minimizing the side effects, and decreasing the risk of recurrence are important goals.

Investigators have attempted to identify predictive factors other than the pathological stage for colorectal cancer recurrence. Both oncogenes, such as c-myc, TGF-*β*, BRAF, and p53, and clinicopathological factors, including lymphatic or venous invasion and budding 6, reportedly predicted recurrence; however, to date, none of these proved to be statistically reliable as predictive factors for use in clinical practice 7. Currently, the glycoproteins CEA and CA19-9 are frequently used to diagnose recurrence or metastasis with colorectal cancer 8–10.

Changes in the glycosylation patterns on cells and specific glycotransferases are reportedly related to cell proliferation, differentiation, tissue adhesion, and carcinogenesis 11. Aberrant glycosylation patterns are associated with human breast 12, prostate 13, thyroid 14, and ovarian 15 cancers as well as human colon cancer 16,17. However, analyzing the structural changes of glycoproteins on cell surfaces has been very difficult because the glycosylation structure is highly susceptible to changes in the extracellular environment. Recently, a novel lectin microarray system was developed to comprehensively analyze glycan profiles using lectins that specifically recognized various sugar moieties 18–21. A recent review also proposed its potential application for the diagnosis of adult T-cell leukemia 22.

Thus, in the present study, we used a lectin microarray system to identify predictive distant recurrence markers from resected specimens of colorectal cancer and assessed their statistical significance.

## Materials and Methods

### Patients and clinical samples

We selected surgical specimens and the medical records of colorectal cancer patients who underwent resection with curative intent from 1997 to 2010 at the Department of Surgery I, Oita University. We excluded patients who had received preoperative chemotherapy/chemoradiotherapy. As a learning set, consecutive stage I–III colorectal cancer patients (*n* = 53) were included in this study. All patients were classified using the UICC-TNM staging system 7. All patients were followed up at our hospital's outpatient clinic for an average of 68 months after surgery. Among these 53 patients, 11 developed recurrences that involved at least one distant organ: three in the liver; two in the lung; two in distant LNs and local; and one each in the liver and peritoneum, lung and adrenal gland, lung and local, and peritoneum.

Independent of our primary set, consecutive stage II colorectal cancer patients (*n* = 55) with a median follow-up of 56 months were included for a validation study. Distant recurrence developed in 12 patients: four in the liver and lung; two each in the liver and lung; two in lungs and peritoneum; one each in the peritoneum and a distant lymph node. Written informed consent was obtained from all the patients.

### Sample preparation

Formalin-fixed, paraffin-embedded sections (10 *μ*m for lectin microarray, 3 *μ*m for lectin staining) of colorectal cancer tissue and normal epithelium in the same clinical specimen were placed on glass slides and deparaffinized. Areas of malignant cells were identified for macrodissection under a magnifying glass after staining with hematoxylin and eosin. In addition, regions that contained normal epithelial tissues were macrodissected.

### Protein extraction and lectin microarray

The lectin microarray procedures were performed according to the manufacturer's protocol. After washing three times with phosphate-buffered saline (PBS), tissue pellets were collected by centrifugation, resuspended with 20 *μ*L of PBS containing 0.5% NP40 (Nonidet P40), and then sonicated with a Bioruptor UCW-310 (Cosmo Bio, Tokyo, Japan). Protein concentrations were determined with a Micro BCA Protein Assay Reagent Kit (Cat No. 23235; Thermo Scientific, Rockford, IL) and diluted to 15 *μ*g/mL with PBS. Glycoprotein fractions (25 ng each) were labeled with Cy3 using a Probing Solution (GP Biosciences Ltd., Tokyo, Japan) in a 100 *μ*L volume, applied to each well of a LecChip™ (GP Biosciences Ltd.), and then incubated in the dark in a chamber (>80% humidity at 20°C) for 15 h. Wells were washed three times with probing buffer before each application to reduce nonspecific background staining. After the binding reaction, fluorescent images of the lectin microarrays were acquired using a GlycoStation™ Reader 1200 evanescent-field fluorescence scanner (GP Biosciences Ltd.). Data were analyzed using GlycoStation™ Tools Pro Suite 1.5. Lectin–glycan interaction (LGI) values were normalized over 45 different lectins by setting the average intensity of the 45 lectins to 100%.

### *β*-actin staining

We performed *β*-actin staining for randomly selected samples (four cancerous and four normal epithelia) for normalization. Tissue sections were deparaffinized and soaked in 0.01 mol/L sodium citrate buffer. A primary antibody against *β*-actin (cat. no. ab8226; Abcam, Cambridge, MA) was used at a 1:500 dilution and staining was developed using a biotin–avidin–peroxidase method. Rabbit serum was used to reduce nonspecific background staining. All sections were counterstained with hematoxylin.

### Lectin staining of colorectal tissue

After deparaffinization using xylene and a graded alcohol series, slides were immersed in diluted Antigen Unmasking Solution (Vector Laboratories, Burlingame, CA) to enhance lectin activity. The slides were transferred to 10 mmol/L citrate buffer (pH 6.0) and heated at 95°C for 40 min. After cooling to room temperature, the sections were washed in PBS (pH 7.5) for 5 min. The glass slides were incubated in methanol containing 0.3% hydrogen peroxide for 10 min to reduce endogenous peroxidase activity. To block endogenous biotin and streptavidin, the glass slides were pretreated by incubating in a solution prepared from four drops of streptavidin solution with 1 mL of Carbo-Free Blocking Solution (1 × concentration) at room temperature for 15 min. After briefly rinsing with PBS, the slides were soaked with four drops of Biotin Blocking Solution at room temperature for 15 min. The sections were then incubated with biotinylated ABA lectin purified from *Agaricus bisporus* lectin (J-OIL MILLS, Tokyo, Japan) used at a concentration of 2 *μ*g/mL for 30 min, incubated with VECTASTAIN® Elite ABC Reagent (Vector Laboratories, Burlingame, CA) for an additional 30 min, and washed in PBS for 5 min. Finally, the reaction products were visualized by incubating the glass slides with diaminobenzidine (DAB) reagent (Vector Laboratories). The sections were counterstained with hematoxylin. Because ABA has dual sugar-binding affinities, Gal*β*1–3GalNAc*α* or GalcNAc), the specificity of ABA binding was tested by adding 10 mmol/L Gal*β*1–3GalNAc*α*-Thr (Tokyo Chemical Industry Co. Ltd, Tokyo, Japan) or *N*-acetyl-d-glucosamine (Sigma, St. Louis, MO), respectively.

### Statistical analysis

The net intensity value for each microarray spot was calculated by subtracting the background value from the raw signal intensity value. Triplicate readings for each lectin were averaged. Signal intensity results were compared between sample groups by a Mann–Whitney test or Welch's *t*-test. Strong correlations (*R*^2^ = 0.999) were found between normalized and non-normalized data sets. Prognostic factors in patient subgroups were compared by a chi-square test. The Kaplan–Meier method was used to generate survival curves, and statistical comparisons of these curves were made using a log-rank test. Multivariate survival analyses were performed using a Cox proportional hazards model. The cutoff point for a particular lectin was determined using receiver operating characteristic (ROC) curve analysis. A *P* value of <0.05 was considered statistically significant, and for the log-rank test a *P* value of <0.01 was considered statistically significant. All analyses were performed using SPSS release 20 (SPSS Inc., Chicago, IL).

## Results

### Lectin microarray profiles of colorectal cancer tissue

Table1 shows the differential glycan analysis results for 45 lectins (LGI values) expressed by cancerous and normal epithelia in colorectal tissues. The following 12 lectins exhibited significantly increased LGI values for cancer tissues compared with those for normal epithelia: PSA, SNA, SSA, TJA-I, NPA, ConA, GNA, HHL, ABA, PWN, MPA, and Calsepa. In contrast, the following 11 lectins exhibited significantly decreased LGI values for binding to cancer tissues: PHA(L), ECA, RCA120, PHA(E), ACG, BPL, TJA-II, WFA, VVA, DBA, and SBA.

**Table 1:** Differential glycan analysis between colorectal cancer tissue (*n* = 53) and normal control (*n* = 53) using data from 45 lectins

Lectin	Normal average	Tumor average	T/N ratio	*P*-value
LTL	17.4	21.6	1.24	N.S.
PSA	53.1	62.0	1.17	<0.005
LCA	68.3	74.1	1.08	N.S.
UEA_I	47.7	54.2	1.14	N.S.
AOL	173.8	170.8	0.98	N.S.
AAL	221.4	211.9	0.96	N.S.
MAL_I	38.2	38.0	1.00	N.S.
SNA	64.9	113.9	1.75	<0.005
SSA	69.7	102.1	1.46	<0.005
TJA-I	114.6	131.2	1.15	0.024
PHA(L)	61.4	44.1	0.72	<0.005
ECA	40.5	32.2	0.79	<0.005
RCA120	178.6	145.3	0.81	<0.005
PHA(E)	158.5	136.9	0.86	<0.005
DSA	256.8	248.3	0.97	N.S.
GSL-II	25.2	29.7	1.18	N.S.
NPA	69.0	106.3	1.54	<0.005
ConA	38.3	49.2	1.28	0.005
GNA	52.6	88.2	1.68	<0.005
HHL	32.8	52.3	1.60	<0.005
ACG	257.3	207.3	0.81	<0.005
TxLC_I	83.4	87.9	1.05	N.S.
BPL	122.5	106.3	0.87	<0.005
TJA-II	176.2	145.6	0.83	<0.005
EEL	32.9	32.0	0.97	N.S.
ABA	67.2	90.2	1.34	<0.005
LEL	313.8	323.9	1.03	N.S.
STL	265.5	271.9	1.02	N.S.
UDA	235.8	242.8	1.03	N.S.
PWM	44.3	59.3	1.34	<0.005
Jacalin	138.9	144.4	1.04	N.S.
PNA	33.4	35.9	1.07	N.S.
WFA	121.3	80.4	0.66	<0.005
ACA	144.6	137.8	0.95	N.S.
MPA	41.5	48.3	1.16	0.016
HPA	67.1	54.7	0.81	N.S.
VVA	32.2	23.2	0.72	<0.005
DBA	75.9	43.2	0.57	<0.005
SBA	76.9	50.6	0.66	<0.005
Calsepa	56.8	72.3	1.27	<0.005
PTL_I	34.2	32.3	0.94	N.S.
MAH	50.8	47.5	0.94	N.S.
WGA	190.0	194.8	1.03	N.S.
GSL_I_A4	37.2	36.6	0.98	N.S.
GSL_I_B4	17.1	17.8	1.04	N.S.

The ratios of tumor versus normal (T/N) were calculated. N.S., not significant; Mann–Whitney *U* test.

### *β*-actin staining

**Figure 1:**
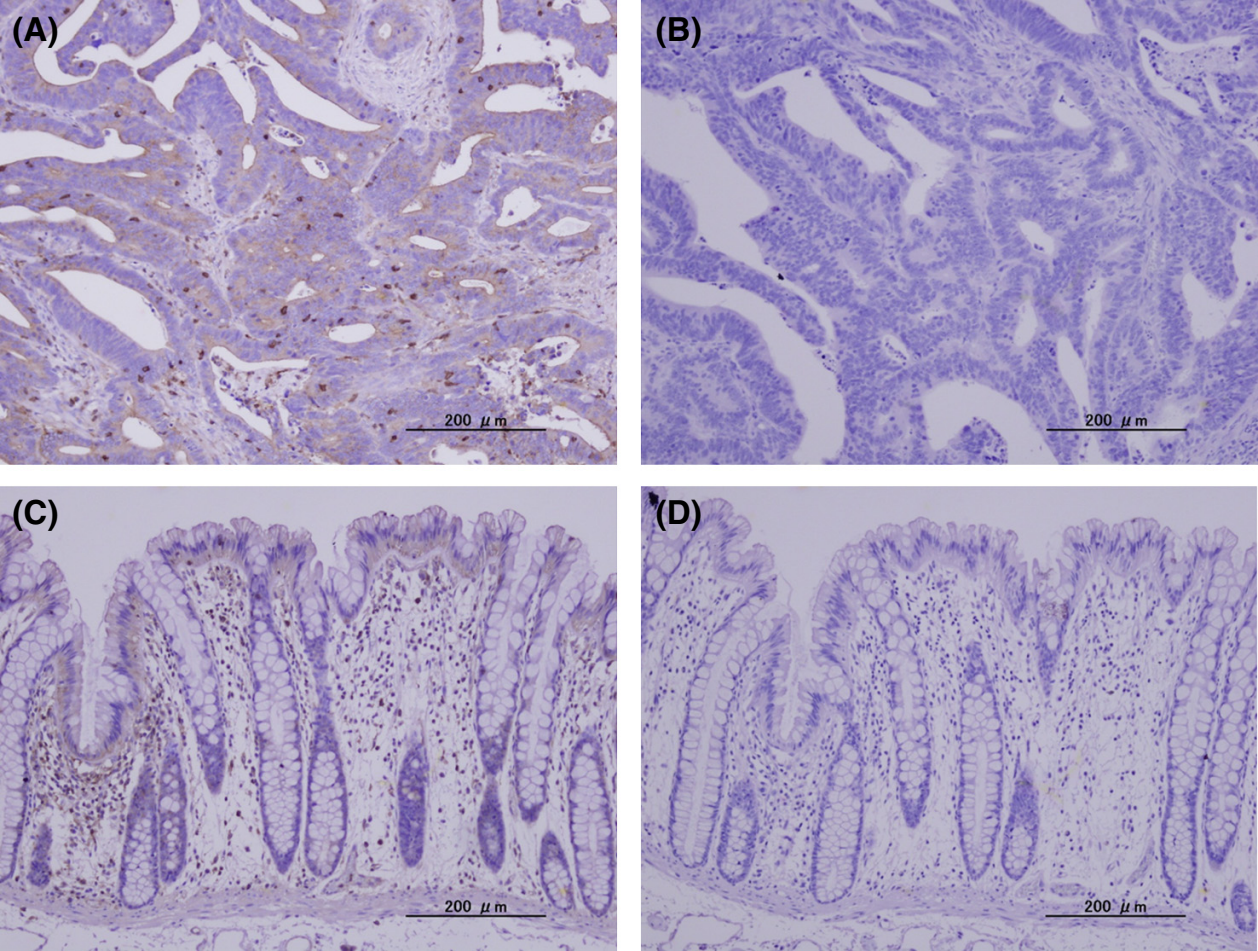
*β*-actin staining. *β*-actin in the colorectal cancer tissues (A) or normal colorectal tissues (C) were visualized with anti-human *β*-actin mAb and biotin–avidin–peroxidase method. Hematoxylin staining of the colorectal cancer tissues (B) or normal colorectal tissues (D) as a control were also performed (×100).

We used *β*-actin staining to establish that the specimens analyzed by lectin microarray had maintained protein molecules both in the membrane and cytosolic fractions. Moderate *β*-actin staining was observed either in the apical surface or the cytoplasm of all cancerous tissues (Fig1A) and lymphocytes or the cytoplasm of surface epithelia of normal mucosa (Fig1C).

### Associations between distant recurrence and clinicopathological factors including lectin signals of postoperative colorectal cancer patients

We examined the clinicopathological factors and lectin expression related to distant recurrence. By univariate analysis, distant recurrence was associated with tumor invasion (*P* = 0.050), the cancer site (*P* = 0.002), and the lymph node status (*P* = 0.023). Of the 45 lectins tested, only HHL- and ABA-binding signals (T/N ratios) were significantly associated with distant recurrence (Table2).

**Table 2:** Correlation between distant recurrence and clinicopathological factors, including lectin signals, in patients who underwent curative resection for stage I–III colorectal cancer

Factors	Category	Recurrence (−)	Recurrence (+)	Univariate analysis	Multivariate *P*-value, relative risk (95% CI)
		(*n* = 42)	(*n* = 11)	*P*-value	
Age, mean ± SD (range)		68 ± 13.0 (40–93)	67 ± 16.5 (26–78)	0.249	
Sex	Male	20	8	0.138	
	Female	22	3		
CEA (mean, ng/mL)	<5	29	6	0.140	
	≥5	8	5		
CA19-9 (mean, U/mL)	<37	32	8	0.598	
	≥37	4	2		
Ileus	No	37	11	0.296	
	Yes	5	0		
Adjuvant chemotherapy	No	31	5	0.143	
	Yes	11	6		
Site	Colon	33	3	0.002	N.S.
	Rectum	9	8		
Tumor invasion	pT1/T2	17	1	0.050	
	pT3/T4	25	10		
Lymph node status	pN0-1	37	6	0.023	N.S.
	pN2-	5	5		
Lymphatic invasion	ly−	20	5	0.898	
	ly+	22	6		
Venous invasion	v−	31	8	0.942	
	v+	11	3		
Histology	tub1/tub2	39	11	1.000	
	por/muc	3	0		
pStage	I	17	1	0.122	
	II(A+B)	10	3		
	III(A+B+C)	15	7		
PSA2.367	<2.367	41	10	0.375	
	≥2.367	1	1		
SNA	<1.956	30	5	0.105	
	≥1.956	12	6		
SSA	<1.650	29	5	0.136	
	≥1.650	13	6		
TJA-I	<0.866	10	0	0.075	
	≥0.866	32	11		
PHA(L)	<0.839	29	4	0.052	
	≥0.839	13	7		
ECA	<0.781	21	2	0.057	
	≥0.781	21	9		
RCA120	<1.068	35	8	0.340	
	≥1.068	7	3		
PHA(E)	<0.664	5	3	0.206	
	≥0.644	37	8		
NPA	<2.434	39	8	0.096	
	≥2.434	3	3		
ConA	<1.929	33	7	0.257	
	≥1.929	9	4		
GNA	<2.053	29	5	0.136	
	≥2.053	13	6		
HHL	<4.00	41	8	0.025	0.001, 1.69 (1.25–2.28)
	≥4.00	1	3		
ACG	<0.951	35	7	0.154	
	≥0.951	7	4		
BPL	<1.522	41	9	0.106	
	≥1.522	1	2		
TJA-II	<0.974	36	7	0.112	
	≥0.974	6	4		
ABA	<2.180	36	4	0.002	0.011, 3.25 (1.31–8.05)
	≥2.180	6	7		
PWM	<1.846	31	5	0.079	
	≥1.846	11	6		
WFA	<0.802	32	8	0.545	
	≥0.802	10	3		
MPA	<2.456	41	9	0.106	
	≥2.456	1	2		
VVA	<2.592	41	10	0.375	
	≥2.592	1	1		
DBA	<0.537	22	4	0.344	
	≥0.537	20	7		
SBA	<0.997	22	4	0.344	
	≥0.997	20	7		
Calsepa	<1.541	31	5	0.079	
	≥1.541	11	6		

**Figure 2:**
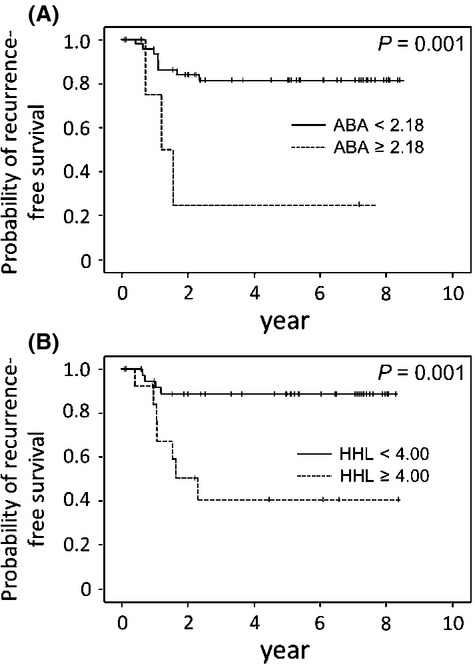
Recurrence-free survival analysis of stage I–III colorectal cancer patients (*n* = 53) with *Agaricus bisporus* (ABA) and *Hippeastrum hybrid* (HHL) status. (A) Five-year recurrence-free survival were 82.0% and 24.7% for the patients with ABA <2.18, and ABA ≥2.18 (*P* = 0.001). (B) Five-year recurrence-free survival were 88.8% and 40.9% for the patients with HHL <4.00, and HHL ≥4.00 (*P* = 0.001).

Multivariate analyses showed that increased HHL- and ABA-binding signals (T/N ratios) were independent predictive factors for worse recurrence-free survival after primary colorectal cancer resection. For HHL: relative risk (RR) = 1.69; 95% confidence interval (CI): 1.25–2.28; *P* = 0.001. For ABA: RR = 3.25; 95% CI: 1.31–8.05; *P* = 0.011 (Table2). Figure2 shows the Kaplan–Meier recurrence-free survival curves with log-rank comparisons based on (A) ABA- and (B) HHL-binding signals. The 5-year recurrence-free survival were 82.0% and 24.7% for patients with ABA of <2.18 and those with ABA of ≥2.18, respectively (*P* = 0.001; Fig2A). The 5-year recurrence-free survival were 88.8% and 40.9% for patients with HHL of <4.00 and those with HHL of ≥4.00, respectively (*P* = 0.001; Fig2B).

As shown in Table1, PHA(L) showed significantly increased LGI values for cancer tissue compared with those in normal epithelia. However, Kaplan–Meier recurrence-free survival curves (Fig. S1) indicated only a marginally significant difference, 84.3% with PHA(L) of <0.839 and 52.9% with PHA(L) of ≥0.893 (*P* = 0.021). Thus, we selected ABA and HHL LGI values for further investigation.

**Figure 3:**
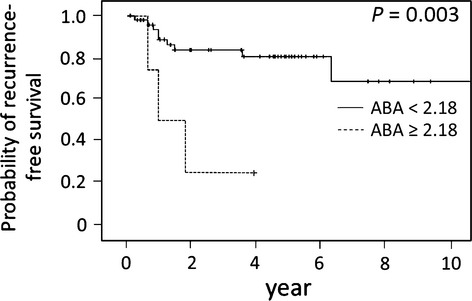
Recurrence-free survival analysis of stage II colorectal cancer patients (*n* = 55) with *Agaricus bisporus* (ABA) status in the validation setting. Recurrence-free survival according to ABA status (*P* = 0.003).

In our validation set, univariate analysis showed that distant recurrence was significantly associated only with ABA-binding signals (T/N ratios) (*P* = 0.029; Table3). Figure3 shows the Kaplan–Meier recurrence-free survival curves with log-rank comparisons based on ABA-binding signals. ABA staining was identified as an independent predictive factor for worse recurrence-free survival after primary colorectal resection.

**Table 3:** Correlation between distant recurrence and clinicopathological factors, including lectin signals, in patients who underwent curative resection for stage II colorectal cancer in the validation setting

Factors	Category	Recurrence (−) (*n* = 43)	Recurrence(+) (*n* = 12)	Univariate analysis *P*-value
Age, mean ± SD (range)		70.3 ± 10.0 (49–93)	74.0 ± 10.4 (53–89)	0.266
Sex	Male	23	9	0.182
	Female	20	3	
CEA (ng/mL)	<5	35	7	0.129
	≥5	8	5	
CA19-9 (U/mL)	<37	40	12	1.000
	≥37	3	0	
Ileus	No	39	10	0.602
	Yes	4	2	
Adjuvant chemotherapy	No	43	11	0.218
	Yes	0	1	
Site	Colon	42	12	1.000
	Rectum	1	0	
Tumor invasion	pT3	42	12	1.000
	pT4	1	0	
Lymphatic invasion	ly−	25	5	0.311
	ly+	18	7	
Venous invasion	v−	36	8	0.230
	v+	7	4	
Histology	tub1/tub2	40	11	1.000
	por/muc	3	1	
HHL	<4.00	28	6	0.503
	≥4.00	15	6	
ABA	<2.180	42	9	0.029
	≥2.180	1	3	

### Lectin staining

**Figure 4:**
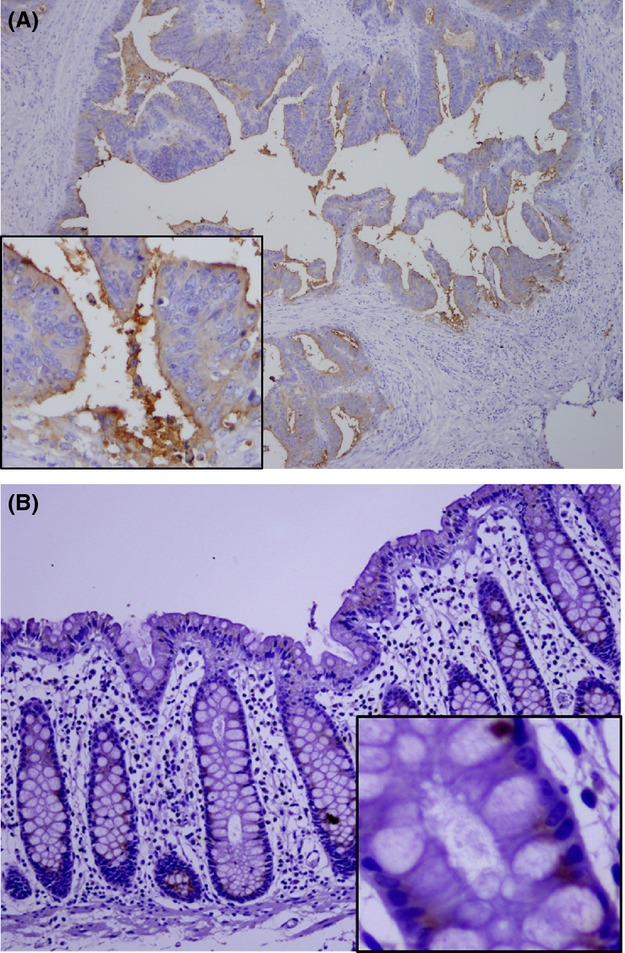
*Agaricus bisporus* (ABA) staining for colorectal cancer tissues. (A) Strong ABA staining was detected within the cytoplasm of cancer cells and the apical surface (×100, ×400). (B) ABA staining was detected in the supranuclear region of the glandular epithelium of normal colorectal tissues (×100, ×400).

We attempted to confirm ABA histological binding using histochemical analysis using biotin-labeled ABA for randomly selected samples (15 cancerous and 15 normal epithelia). There was a high frequency of strong ABA staining in the cytoplasm and moderate staining in apical surfaces of cancerous tissues (Fig.4A), whereas ABA staining was minimally detected in the supranuclear regions of glandular epithelium in normal mucosa (Fig.4B). For all of these cases, staining was inhibited or greatly diminished after absorption with Gal*β*1–3GalNAc*α*-Thr or *N*-acetyl-d-glucosamine.

## Discussion

In this study, we evaluated multiple glycan expression profiles using a lectin microarray system for the tissues of curatively treated subjects, to identify predictive markers for distant colorectal cancer recurrence. The clinicopathological factors and 45 lectins in two independent cohorts showed that an increased ABA-binding signal could be a predictive factor for distant colorectal cancer recurrence (Figs.2A and 3). This is the first report to show that LGI values obtained from a lectin microarray could be used to identify predictive biomarkers for distant colorectal cancer recurrence, and that increased ABA-binding signals were associated with distant recurrence in these patients.

Although multiple methods, such as enzyme-linked immunosorbent assay (ELISA), liquid chromatography, and mass spectrometry, have been used to analyze glycans on the surfaces of cancerous cells, the disadvantages of these methods, including their relatively low sensitivity, low throughput, labor-intensive sample preparation, and their requirements of specialized knowledge and skills, has hindered their widespread use for diagnosis 23. In comparison, the lectin microarray system used in the present study provides comprehensive, highly reproducible glycan expression profiles and requires only basic biochemical techniques. The 45 independent LGI values could be obtained from formalin-fixed clinical specimens 15. Although this method requires meticulous sample preparation, simultaneous, quantitative profiling of *N*- and *O*-linked glycans (LGI values) can easily be achieved with this system.

Histochemical analysis has shown that there is regionally variable glycan expression in the colon 24. However, it is not known whether the comorbidity that occurs in obstructive colitis with ileus or diabetes mellitus patients can affect the glycan structures of the colorectal epithelium 25. To confirm the results of lectin microarray analysis, which indicated increased ABA LGI values in the cancerous tissues of colorectal cancer subjects, we used ABA lectin staining and detected strong, concomitant signals both on the surfaces and in the cytoplasm of cancerous tissues (Fig.4A). In contrast, ABA staining was weak and limited to lymphocytes and on the surfaces or glandular parts in normal epithelium (Fig.4B). Because we noted that each LGI value varied among normal specimens, we decided to employ a tumor/normal ratio for each clinical specimen to evaluate these values.

Because the available amount of a particular glycoprotein fraction was limited, we adjusted each fraction's concentration and each LGI value was normalized by setting the average of the 45 independent LGI values to 100%. In addition, we used *β*-actin staining to establish that each sample contained proteins (Fig.1).

In this study, ABA was identified as a predictive factor for distant recurrence and was shown to be a prognostic factor. ABA reportedly has two distinct binding sites: Gal*β*1–3GalNAc*α* (TF antigen) and *N*-acetylgalactosamine 26. The TF antigen appears on tumors, such as those in lung cancer 27, bladder carcinoma 28, and colon cancer 29. Langkilde et al. reported that high-level TF antigen expression was associated with poor prognoses for patients with bladder carcinoma 28. Hanish et al. reported that TF antigen expression was associated with tumor progression and metastasis in cells or tissues 30. Cao et al. found that TF antigen expression on primary colon carcinoma was associated with an enhanced risk of liver metastases 31. The results in these previous reports are consistent with our results.

Shigeoka et al. found that treating colon 26 cells with neuraminidase resulted in TF antigen exposure and consequently a higher frequency of liver metastases in an animal model. In addition, they showed that metastasis to the liver could be prevented by an antibody directed against the TF antigen 32. An antibody against the TF antigen may have a possible therapeutic effect for prophylaxis against metastasis to the liver.

The American Society of Clinical Oncology stated that patients with stage II disease could be considered for adjuvant therapy, including those with inadequately sampled nodes, T4 lesions, perforation, or poorly differentiated tumors 3. The European Society for Medical Oncology guidelines recommended that patients with stage II disease should be considered for adjuvant therapy in cases that included <12 lymph nodes, vascular invasion, lymphatic invasion, perineural invasion, poorly differentiated tumors, obstruction, perforation, or a pT4 tumor 33. In addition to these clinicopathological factors, previous studies have investigated the potential of various molecular and genetic biomarkers, including MSI 34, LOH18q 35, p53 36, KRAS 37, and BRAF 38, for better patient selection and classification for adjuvant therapy. However, no appropriate biomarker has yet been identified to decide the adjuvant therapy for stage II colorectal cancer patients.

In this study, using a lectin microarray, we showed that ABA intensity measurements could be an effective predictive biomarker for distant colorectal cancer recurrence. This system has significant advantages, including high-throughput performance with simple pretreatments, ultrasensitivity, and high reproducibility. However, various factors that could affect the glycan structure of the normal epithelium, such as tumor location, presence or absence of obstructive colitis, and diabetes mellitus, need to be clarified. In addition, lectin profiling analysis of different types of lesions is required because cancer tissue heterogeneity may affect its lectin-binding profile.

The major limitation of the current study was the relatively small number of events available for analysis, and other candidates may have been overlooked because of a lack of statistical significance. Nevertheless, those factors that were shown to be significant in a validation set are worth considering in further research. Experiments using cell lines or animal models should be developed to verify whether or not this altered ABA binding could actually induce metastases.

In conclusion, our results indicated that the tumor/normal ratio for ABA binding could be a novel predictive biomarker for distant recurrence of curatively resected colorectal cancer.

## Supporting Information

**Figure S1.** Recurrence- free survival curve of stage I–III colorectal cancer patients (n=53) with PHA(L) status.
