# Supplementary figures and images for: Establishment of new predictive markers for distant recurrence of colorectal cancer using lectin microarray analysis
Source: Cancer Med. 2014 Oct 30;4(2):293–302. doi: 10.1002/cam4.342 (PMC4329012; doi:10.1002/cam4.342)

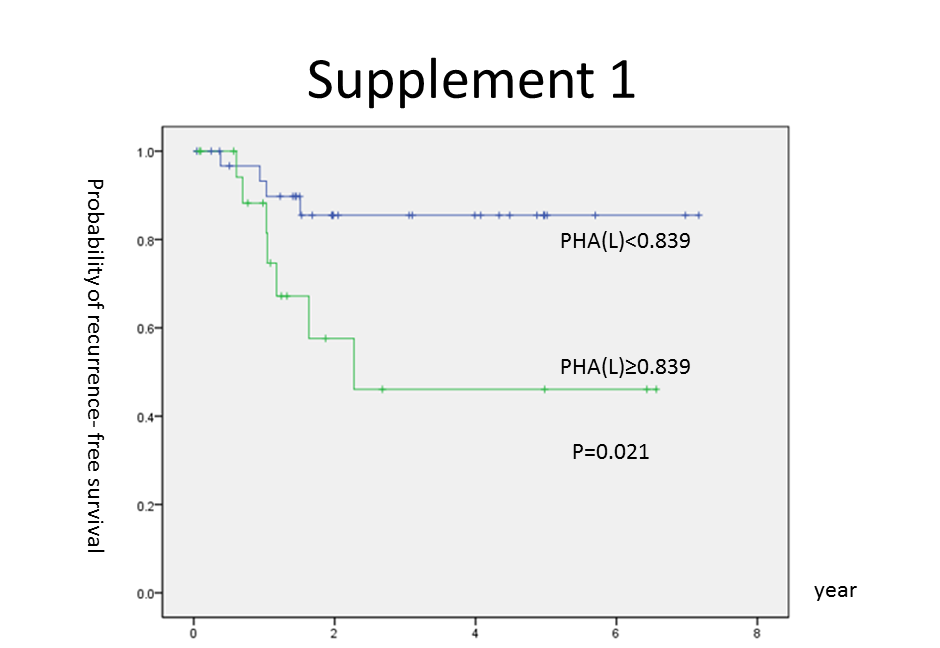

Supplement: Supplementary file 1 [file cam40004-0293-sd1.tif]
